# Optimized therapeutic potential of Sijunzi-similar formulae for chronic atrophic gastritis via Bayesian network meta-analysis

**DOI:** 10.17179/excli2024-7618

**Published:** 2024-09-06

**Authors:** Meilan Huang, Shiman Luo, Jiayue Yang, Huiling Xiong, Xiaohua Lu, Xiao Ma, Jinhao Zeng, Thomas Efferth

**Affiliations:** 1 Department of Gastroenterology, Hospital of Chengdu University of Traditional Chinese Medicine, Chengdu 610072, China; 2 School of Clinical Medicine, Chengdu University of Traditional Chinese Medicine, Chengdu 610075, China; 3 State Key Laboratory of Southwestern Chinese Medicine Resources, School of Pharmacy, Chengdu University of Traditional Chinese Medicine, Chengdu 611137, China; 4 Department of Pharmaceutical Biology, Institute of Pharmaceutical and Biomedical Sciences, Johannes Gutenberg University, 55128 Mainz, Germany; 5 TCM Regulating Metabolic Disease Key Laboratory of Sichuan Province, Hospital of Chengdu University of Traditional Chinese Medicine, Chengdu 610072, China

**Keywords:** chronic atrophic gastritis, Sijunzi-similar formulae, drug integration, Bayesian network meta-analysis

## Abstract

Chronic atrophic gastritis (CAG) is considered as a significant risk factor for triggering gastric cancer incidence, if not effectively treated. *Sijunzi* decoction (SD) is a well-known classic formula for treating gastric disorders, and *Sijunzi*-similar formulae (SF) derived from SD have also been highly regarded by Chinese clinical practitioners for their effectiveness in treating chronic atrophic gastritis. Currently, there is a lack of meta-analysis for these formulae, leaving unclear which exhibits optimal efficacy. Therefore, we employed Bayesian network meta-analysis (BNMA) to evaluate the efficacy and safety of SF as an intervention for CAG and to establish a scientific foundation for the clinical utilization of SF. The result of meta-analysis demonstrated that the combination of SF and basic therapy outperformed basic therapy alone in terms of clinical efficacy rate, eradication rate of H. pylori, and incidence of adverse events. As indicated by the SUCRA value, *Chaishao Liujunzi* decoction (CLD) demonstrated superior efficacy in enhancing clinical effectiveness and ameliorating *H. pylori *infection, and it also showed remarkable effectiveness in minimizing the occurrence of adverse events. Comprehensive analysis of therapeutic efficacy suggests that CLD is most likely the optimal choice among these six formulations, holding potential value for optimizing clinical treatment strategies.

See also the graphical abstract(Fig. 1).

## Introduction

Chronic atrophic gastritis (CAG), characterized by inherent glandular atrophy of the gastric mucosa, is a digestive disorder primarily caused by factors such as *Helicobacter pylori* infection, autoimmune reactions, and alcoholism (Shah et al., 2021[47]). The damage to the gastric mucosa resulting from various factors leads to chronic inflammation, CAG, intestinal metaplasia, and subsequent dysplasia, which are widely acknowledged as fundamental processes in the development of intestinal-type gastric cancer (Correa, 1992[8]). CAG is regarded as a critical stage in this progression (Zhang et al., 2020[74]). Globally, the prevalence of atrophic gastritis is concerning, with epidemiological studies revealing that the prevalence of CAG is approximately 25 % and has shown a noticeable upward trend from 2010 to 2020 (Lv et al., 2024[37]). More recently, a study revealed that over the past fifty years, the prevalence of CAG among precancerous lesions of gastric cancer has remained persistently high across the Asian continent (Li et al., 2024[28]). This implies that without effective intervention, there will be an increase in the number of gastric cancer patients in the future, posing significant health risks to individuals and imposing substantial societal burdens (Yang et al., 2022[65]).

Currently, the relevant treatment strategies remain imperfect. Drug therapy serves as the primary approach to manage the disease. The commonly employed options include triple and quadruple therapy, which typically entail the use of a proton pump inhibitor, two different classes of antibiotics, and/or a bismuth agent. These treatment regimens focus on eradicating *H. pylori*-induced infection, safeguard the gastric mucosa, and enhance gastric motility (Møller et al. 2011[39]; Lahner et al. 2018[21]). However, over time, increasing limitations and issues have been uncovered. Prolonged utilization of proton pump inhibitors has the potential to disrupt the balance of intestinal microbiota, thus elevating the susceptibility to gastrointestinal infections (Freedberg et al., 2015[11]). On the other hand, patients with CAG are more prone to infection by drug-resistant strains, posing challenges to their treatment (Yang et al. 2022[65]; Shao et al. 2024[48]).

*Sijunzi* decoction (SD), a classical formula with a nearly thousand-year history for treating stomach diseases, consists of four herbal ingredients: *Panax ginseng *rhizome, *Atractylodes macrocephala* Koidz rhizome, Roasted Licorice, and *Poria cocos*. Various modified prescriptions derived from SD, such as *Liujunzi* decoction (LD), *Chaishao Liujunzi* decoction (CLD) and *Shenling Baizhu* powder (SBP), are also utilized by Chinese clinicians in the treatment of CAG (Supplementary Table 1). Several studies have reported the positive effects of *Sijunzi*-similar formulae (SF) on improving gastric mucosal inflammation, promoting gastric mucosal repair, controlling *H. pylori* infection, and reducing symptoms such as abdominal pain and bloating (Gan et al., 2017[12]; Tian et al., 2019[52]; Lv et al., 2017[38]). However, conclusive evidence is lacking to prove the superiority of SF compared to conventional therapies. It also remains unclear which formula is most effective within this category.

**Figure 1:**
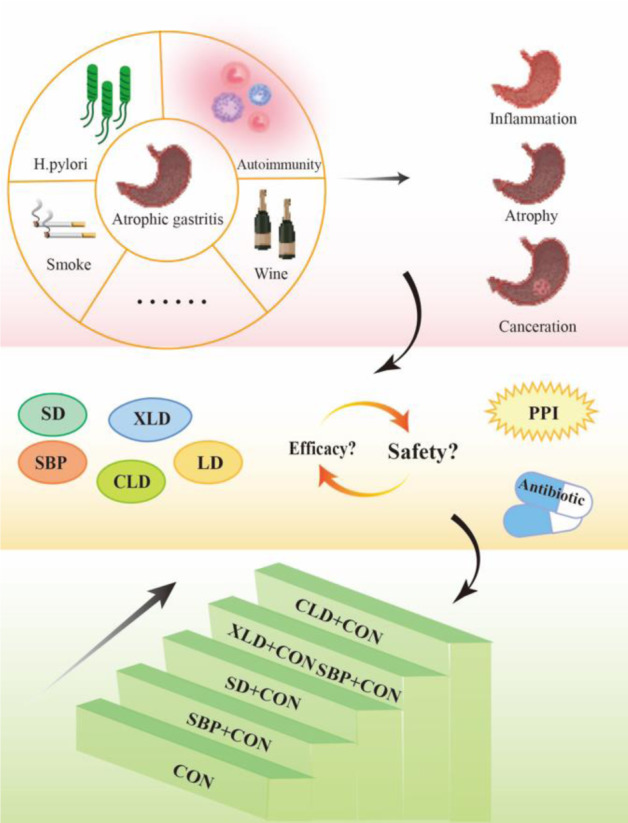
Graphical abstract Notes: SD, *Sijunzi *decoction. LD, *Liujunzi* decoction. XLD, *Xiangsha Liujunzi* decoction. SBP, *Shenling Baizhu* powder. CLD, *Chaishao Liujunzi* decoction. CON, Conventional therapy

Therefore, the objective of this study was to compare the efficacy and safety of different types of formulas using Bayesian network meta-analysis (BNMA), with the aim of providing further insights into the selection of optimal treatments for CAG (Figure 1(Fig. 1)).

## Materials and Methods

In accordance with the Cochrane Handbook and the Preferred Reporting Items for Systematic Reviews and Meta-Analyses (PRISMA) statement, this BNMA adheres to the criteria and is registered with PROSPERO (CRD42023420474) (https://www.crd.york.ac.uk/PROSPERO).

### Search strategy

Seven databases (PubMed, EMBASE, Cochrane Library, Web of Science, Chinese National Knowledge Infrastructure (CNKI), the VIP Medicine Information System and the Wan fang Database) were searched to identify studies on the treatment of CAG with SF. The searches were conducted from database creation to March 2024. The keyword composition included “chronic atrophic gastritis”, “*Sijunzi* decoction”, “*Liujunzi* decoction”, “*Xiangsha Liujunzi* decoction”, “*Chaishao Liujunzi* decoction” and “*Shenling baizhu* powder”. In order to ensure a thorough search, our search strategy was executed twice. Initially, we combined the term chronic atrophic gastritis with the five subsequent keywords. For the second search, the same keywords were used together to ensure that no eligible studies were missed during the initial search. Supplementary Table 2 displays the search strategy used in English database. Similar search strategies were employed in the other databases as well.

### Inclusion and exclusion criteria

The study inclusion criteria were determined based on the PICOS principle, considering the following aspects: (i) Study Type: the literature clearly stated that the study type were clinical randomized controlled trials. (ii) Subjects: patients with CAG who meet the norms for the diagnosis and treatment specifications of CAG (Banks et al., 2019[2]). There were no notable distinctions in the general data (gender, age, disease duration and degree of gastric mucosal atrophy, etc.) (iii) Interventions: The experimental group received a combination of conventional therapy along with one of the SF herbal formulas, whereas the control group adopted solely conventional therapy, which consist of “*Sijunzi* decoction + conventional therapy” (SD+CON), “*Liujunzi* decoction + conventional therapy” (LD+CON), “*Xiangsha* Liujunzi decoction + conventional therapy” (XLD+CON), “*Chaishao Liujunzi* decoction + conventional therapy” (CLD+CON), “*Shenling baizhu* powder + conventional therapy” (SBP+CON) and “conventional therapy” (CON). (iv) Evaluation indexes: clinical efficiency, *H. pylori* eradication rate, adverse reaction occurrence report and TCM symptom score. (v) All studies included in the analysis featured at least one outcome indicator. 

The following studies were excluded: (i) The type of article is a review, meta-analysis, animal experiment, dissertation, or observational paper. (ii) No mention of randomized controlled trials or RCTs in the literature. (iii) Interventions with other Chinese patent medicine or herbal prescriptions. (iv) Unclear or incomplete outcome indicator.

### Data collection

To begin with, all documents retrieved through the search strategy are imported into Endnote X9, followed by a comprehensive check for duplicates utilizing both computerized algorithms and manual inspection to ensure their elimination. Subsequently, a meticulous screening process is employed to exclude literature deemed irrelevant to the study. The remaining literature is then downloaded for in-depth evaluation. A rigorous assessment is undertaken, adhering to the established inclusion and exclusion criteria, to ascertain the final selection for inclusion in the study.

We extracted the following elementary data from the included documents: (i) The last name of the first author and the publication year. (ii) Participant count in both the experimental and control groups. (iii) The mean age, gender, and disease duration of patients in the experimental and control groups. (iv) Treatment intervention and duration in both the experimental and control groups. (v) Outcome indicator: Clinical efficiency, *H. pylori* eradication rate, adverse reaction occurrence report and TCM symptom score. The outcome indicators were evaluated individually based on specific criteria: (i) Clinical efficiency: Clinical symptoms such as stomach pain significantly improved or disappeared, gastroscopic reduction of mucosal lesions by more than 50 % and obvious relief or disappearance of inflammation. If there is no significant improvement or even a worsening of CAG-related symptoms or signs, and less than 50 % reduction of gastric mucosal atrophy lesions or even higher than the original is considered ineffective. (ii) *H. pylori* eradication rate: *H. pylori* infection is considered eradicated when there is a negative test result obtained from a ^14^C-urea breath test or ^13^C breath test, sinus tissue section staining, or sinus mucosal rapid urease test breath test. (iii) Symptom Score: Scored according to the severity of abdominal pain and loss of appetite on a scale of 3 as the highest score. (iv) Adverse reaction occurrence report: Includes adverse reactions such as nausea, vomiting, rash and dizziness.

### Assessment of study quality and reporting

Our team utilized Review Manager 5.3 software to assess the methodological rigor of the literature incorporated in our study. The risk of bias assessment tool recommended in the Cochrane Systematic Assessor's Handbook 5.1.0, which includes seven assessment areas: randomization, allocation concealment, blinding, completeness of outcome data, selection of reports, and other possible sources of bias. To evaluate each area, we assigned ratings of high risk, low risk, or unclear risk based on the actual situation of the study.

### Data processing strategy

The data were statistically processed and analyzed using R4.4.0 and Stata15.0 software. Different analysis methods were employed based on the types of outcome indicators. To assess outcomes such as clinical efficacy,* H. pylori* eradication, and the incidence of adverse effects, we calculated the relative risk (RR) and the corresponding 95 % confidence intervals (CIs) to measure the impact of the intervention. Conversely, for continuous variables such as scores for abdominal pain and loss of appetite symptoms, we used the standard mean difference (SMD) to calculate the effect size, taking into account differences in units, along with a 95 % CI.

Stata 15.0 software was utilized to map the network evidence for the different outcome indicators, reflecting the basic characteristics of each individual study. BNMA was accomplished using the Markov chain-Monte Carlo method via R4.4.0 software (iteration number set to 60,000). The potential scale reduction factor (PSRF) is used to reflect the convergence, when the PSRF approaches or equals 1, indicating excellent convergence, the consistency model is selected for analysis, while in the case of poor convergence, the inconsistency model is chosen for analysis.

The software R4.4.0 was utilized for ranking the data based on the size of the surface under the probability cumulative ranking curve (SUCRA). The magnitude of *H. pylori* eradication and clinical efficiency showed a positive correlation with the degree of benefit, so Rank 1 indicates the best and Rank N indicates the worst. On the contrary, the incidence of adverse effects and the TCM symptom score were negatively correlated with the degree of benefit, so that Rank 1 indicated the worst and Rank N the best.

## Results

### Included study basic characteristics

**Figure 2:**
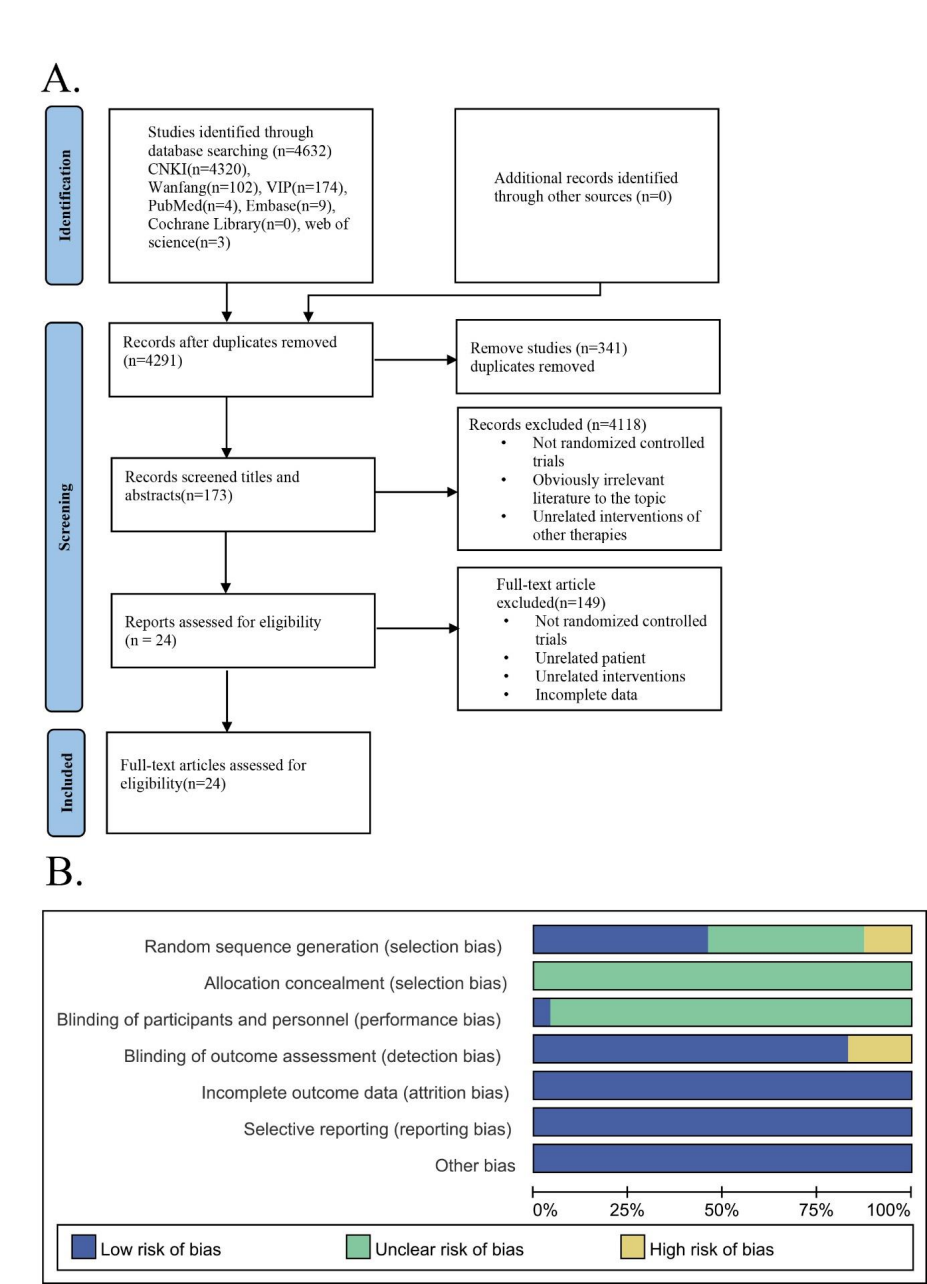
The literature retrieval and Bias Risk Assessment. A: PRISMA flow diagram. B: The risk assessment graphs for each included study

A total of 4320 potentially eligible documents were retrieved from seven databases using the search strategy. The retrieved documents were imported into Endnote X9 software, and a total of 341 duplicates were eliminated through computerization as well as manual review. We carefully examined the title, abstract, and keywords of these documents to exclude 4118 articles that were clearly unrelated to the study's purpose, including reviews, observational studies, meta-analyses, and other irrelevant material. Subsequently, the remaining 173 studies were downloaded and by reading the full text meticulously, we removed literature that did not mention randomized controlled trials, had subjects unrelated to this study and incomplete outcome indicators. Ultimately, this meta-analysis included 24 studies (Figure 2A(Fig. 2)). Table 1(Tab. 1) (References in Table 1: Chen, 2019[6]; Chen et al., 2018[7]; Hu, 2020[16]; Li, 2018[26], 2020[25]; Li and Gang, 2016[27]; Lin, 2020[29]; Liu, 2014[33]; Liu and Chen, 2020[31]; Lu, 2015[34]; Luo, 2019[36]; Peng, 2016[43]; Shen, 2016[49]; Wang, 2021[55]; Wang et al., 2019[57]; Xie, 2019[59]; Xie and Li, 2022[58]; Xu, 2014[61]; Xu et al., 2010[63]; Yan, 2019[64]; Yin and Xu, 2021[70]; Zhao, 2022[76]; Zhou et al., 2015[79]) shows the fundamental features exhibited by the included studies.

### The risk of bias assessment

As recommended by the Cochrane Handbook for Systematic Reviews of Interventions version 5.1.0, we utilized the Review Manager 5.3 software to assess the fulfilled studies for potential bias risks. This part was conducted independently by two members of the research team, Luo and Yang. The resolution of any discrepancies involved a discussion between two team members in order to reach a consensus. If consensus could not be reached, the third member of the research team has been consulted for the purpose of resolving the differences through group discussion and consensus-building (Figure 2B(Fig. 2)). 

Selection bias: Eleven studies using standard randomization methods (random number tables, computer-generated random numbers, etc.) were assessed as having a low risk. Ten studies only mentioned the randomization grouping method but did not provide a specific description, so they were assessed as having an unclear risk. One study grouped patients according to their treatment regimens, and two studies grouped patients according to time of admission order, with a single number being the control group and a double number being the experimental group, so they were assessed as having a high risk.

Allocation and performance bias: The majority of the reviewed studies were rated as “unclear risk” due to insufficient description of their allocation methods. However, one study explicitly mentioned the implementation of a double-blind method, which resulted in a low risk of bias assessment. In contrast, the remaining 23 studies did not provide clear information about the use of blinding for trial participants and personnel, which resulted in an unclear risk of bias rating.

Attrition bias: No studies had missing data or subjects, and all study outcome data were complete, resulting in a low risk assessment for all studies.

Reporting bias: All studies reported predetermined outcome indicators, with no selective reporting of outcomes, and were assessed as having a low risk.

Other bias: No study mentioned any other potential sources of bias, which were rated as “low risk”.

### The outcome of indicator

#### Clinical efficacy

**Figure 3:**
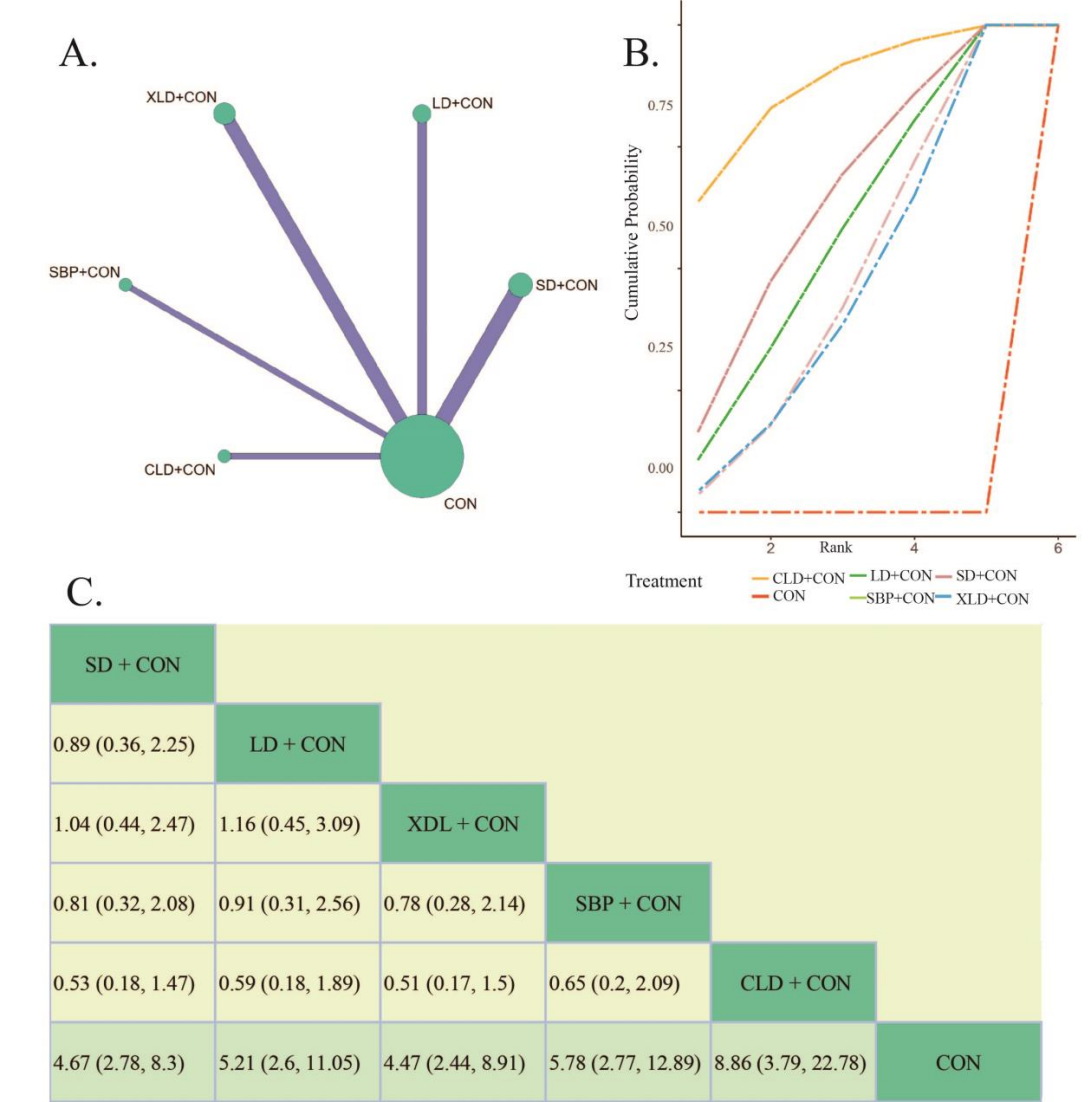
The Bayesian Network Meta analysis of clinical efficiency. A: Network comparisons of clinical efficiency. B: The cumulated probability rank of clinical efficiency. C: League table of Bayesian network meta-analysis for clinical efficiency. (Notes: SD, *Sijunzi *decoction. LD, *Liujunzi* decoction. XLD, *Xiangsha Liujunzi* decoction. SBP, *Shenling Baizhu* powder. CLD, *Chaishao Liujunzi* decoction. CON, Conventional therapy)

Clinical efficacy, commonly assessed through evaluating patients' symptoms, signs, and the degree of improvement in gastric mucosa among other aspects, provides a comprehensive reflection of patients' improvement. This is precisely, why we have chosen it as one of our outcome indicators. 23 randomized controlled trials including 2015 patients with CAG reported on this indicator, which covered all interventions (Figure 3A(Fig. 3)). The test of heterogeneity indicated statistical significance in the variation between studies, land a random effects model was chosen (*P* < 0.00001, *I² *= 78.9 %). Besides, the inconsistency test using the node partitioning method shows that the PSRF is 1, indicating convergence of the iterations well.

We utilize SUCRA to delineate the varied cumulative probabilities associated with each potential ordering of intervention plans, wherein higher SUCRA values connote enhanced efficacy of the treatment regimen. Considering clinical efficacy, the SUCRA results indicated the following order of interventions: CLD+CON (89.37 %) > SBP+CON (69.81 %) > LD+CON (63.81 %) > SD+CON (55.96 %) > XLD+CON (54.38 %) > CON (16.67 %) (Figure 3B(Fig. 3)). The BNMA results demonstrated that compared to the conventional treatment combination (CON), SD+CON (RR= 4.67, 95 % CI 2.78, 8.3), LD+CON (RR= 5.21, 95 % CI 2.6, 11.05), XLD+CON (RR= 4.47, 95 % CI 2.44, 8.91), SBP+CON (RR= 5.78, 95 % CI 2.77, 12.89), and CLD+CON (RR= 8.86, 95 % CI 3.79, 22.78) significantly improved the clinical efficacy rate of CAG (Figure 3C(Fig. 3)). Additionally, the comparison of each of the five interventions of the herbal formula with the conventional treatment combination was not statistically significant. 

From the results above, it is clear that the clinical efficiency of each combination herbal formula group was obviously better than the conventional intervention plan. Furthermore, the strategy of CLD+CON (rank 1) and SBP+CON (rank 2) may represent more ideal approaches for improving clinical efficacy in CAG.

#### H. pylori eradication rate

**Figure 4:**
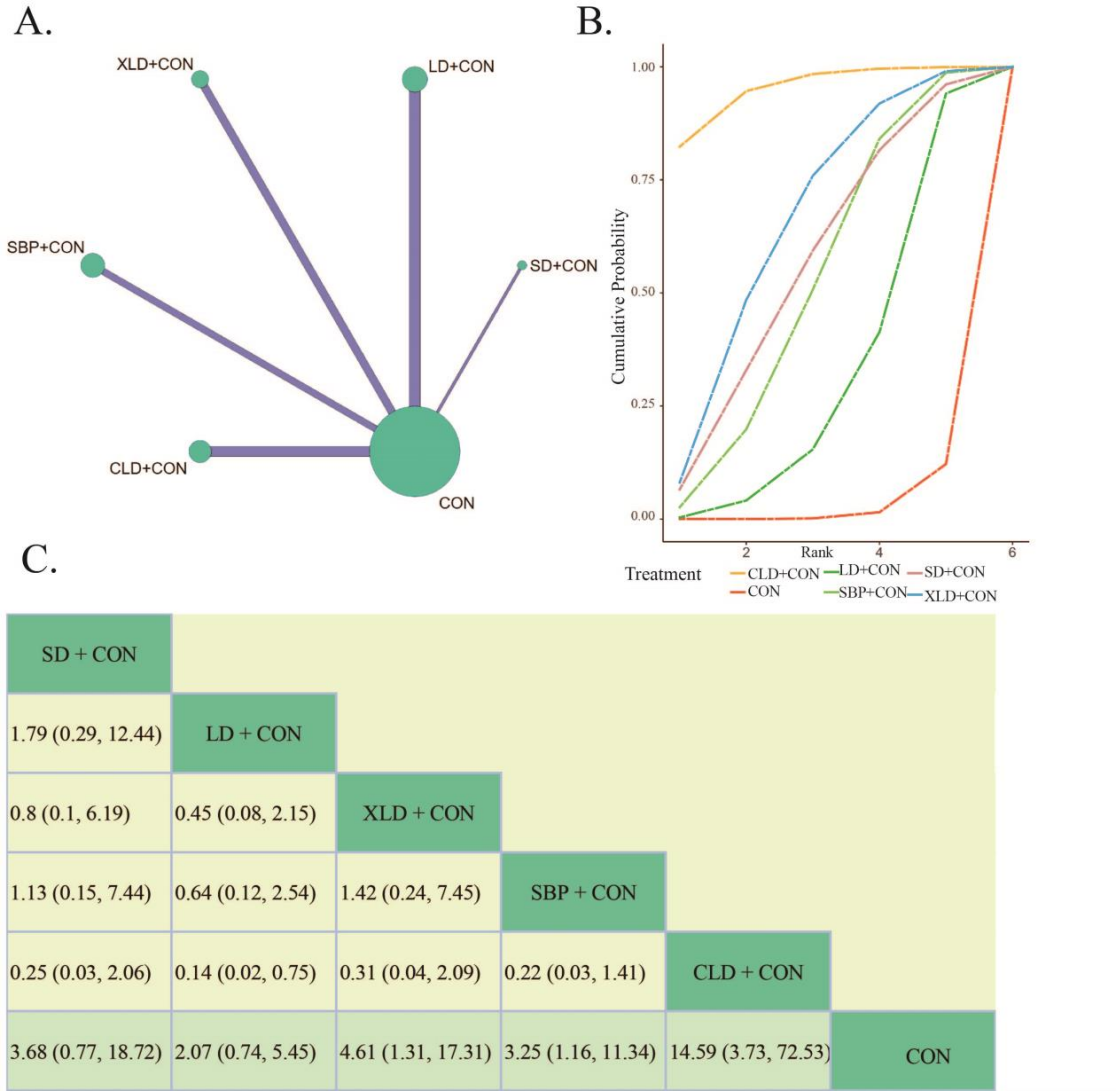
The Bayesian Network Meta analysis of *H. pylori* eradication rate. A: Network comparisons of *H. pylori* eradication rate. B: The cumulated probability rank of *H. pylori* eradication rate. C: League table of Bayesian network meta-analysis for *H. pylori* eradication rate. (Notes: SD, *Sijunzi* decoction. LD, *Liujunzi *decoction; XLD, *Xiangsha Liujunzi *decoction. SBP, *Shenling Baizhu* powder. CLD, *Chaishao Liujunzi* decoction. CON, Conventional therapy)

Globally, *H. pylori* infection is considered one of the major causative factors in atrophic gastritis, which is why we chose *H. pylori* eradication rate as an outcome indicator (Zheng et al., 2024[77]). Ten studies reported *H. pylori* eradication rate in their results, which including 867 *H. pylori*-positive patients (Figure 4A(Fig. 4)). The test of heterogeneity indicated statistical significance in the variation between studies, land a random effects model was chosen (*P*< 0.0001, *I²*= 73.1 %). In addition, the inconsistency test using the node partitioning method shows that the PSRF is 1, indicating convergence of the iterations well.

For *H. pylori* eradication rate, the SUCRA was ranked from highest to lowest: CLD+CON (79.14 %) > XLD+CON (53.90 %) > SD+CON (46.10 %) > SBP+CON (42.67 %) > CON (2.30 %) (Figure 4B(Fig. 4)). Consistently, CLD+CON stands out at *H. pylori* eradication rate. The BNMA results show that compare to CON, XLD+CON (RR= 4.61, 95 % CI 1.31, 17.31), SBP+CON (RR= 3.25, 95 % CI 1.16, 11.34), CLD+CON (RR= 14.59, 95 % CI 3.73, 72.53) could enhance the CAG *H. pylori* eradication rate, and SD+CON (RR= 3.68, 95 % CI 0.77, 18.72), LD+CON (RR= 2.07 , 95 % CI 0.74, 5.45) showed no notable difference in the *H. pylori* eradication rate of CAG (Figure 4C(Fig. 4)). In addition, the comparison of each of the five interventions of the herbal formula with the conventional treatment combination was not statistically significant. 

From the results above, it is clear that the *H. pylori* eradication rate of each combination herbal formula group was obviously better than the conventional treatment intervention plan. The strategy of CLD+CON (rank 1) and XLD+CON (rank 2) may have a relative advantage in treating *H. pylori* eradication rate in CAG.

#### Incidence of adverse effects

**Figure 5:**
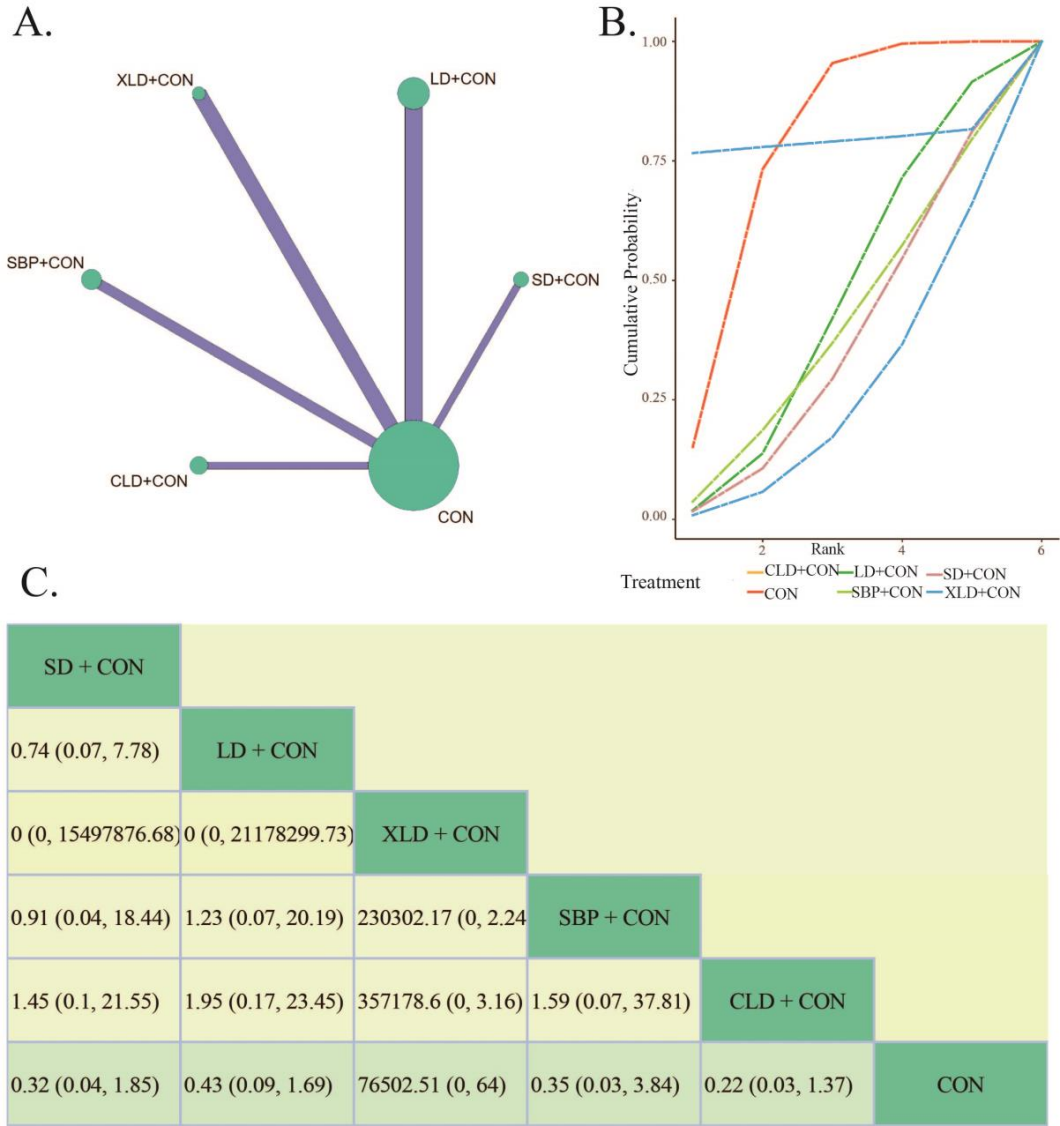
The Bayesian Network Meta analysis of Incidence of adverse effects. A: Network comparisons of Incidence of adverse effects. B: The cumulated probability rank of Incidence of adverse effects. C: League table of Bayesian network meta-analysis for Incidence of adverse effects. (Notes: SD, *Sijunzi *decoction. LD, *Liujunzi* decoction. XLD, *Xiangsha Liujunzi* decoction. SBP, *Shenling Baizhu* powder. CLD, *Chaishao Liujunzi* decoction. CON, Conventional therapy)

Incidence of adverse effects is an essential indicator of drug safety and tolerability, and therefore we used it as an outcome indicator to reflect the safety of these six interventions (Rockhold, 2017[46]). There were 12 studies reporting this outcome indicator with the six interventions. Among these 1050 patients, a total of 62 experienced adverse events such as nausea, vomiting, rash, and vertigo occurred (Figure 5A(Fig. 5)). The results of the heterogeneity test demonstrated no statistically significant differences between studies for the outcome indicator of incidence of adverse reactions (*P *= 0.701, *I²*= 0.0 %). The inconsistency test using the node partitioning method shows that the PSRF is 1, indicating convergence of the iterations well.

The results of ranking the interventions by SUCRA showed that XLD+CON (82.56 %) > CON (80.57 %) > LD+CON (53.48 %) > SBP+CON (49.37 %) > SD+CON (46.26 %) > CLD+CON (37.75 %) (Figure 5B(Fig. 5)). The BNMA results show that compare to CON, SD+CON (RR= 0.32, 95 % CI 0.04, 1.85), LD+CON (RR= 0.43, 95 % CI 0.09, 1.69), XLD+CON (RR= 76502.51, 95 % CI 0, 6.45), SBP+CON (RR= 0.35, 95 % CI 0.03, 3.84), CLD+CON (RR= 0.22, 95 % CI 0.03, 1.37) indicated no notable difference in the incidence of adverse effects (Figure 5C(Fig. 5)). In addition, the comparison of each of the five interventions of the herbal formula with the conventional treatment combination was not statistically significant.

The above results suggest that the group mostly using combined herbal formulations, significantly outperformed the group receiving conventional interventions in terms of the incidence of adverse effects. The strategy of CLD+CON (rank 6) and SD+CON (rank 5) may have a relative advantage in incidence of adverse effects in CAG.

#### Chinese Medicine Symptom Score

When identifying diseases and judging the effectiveness of treatment, TCM will tend to start with the symptoms of patients. The amelioration in patient symptoms and signs is regarded as one of the primary criteria guiding physicians in subsequent pharmacological decisions. Patients with CAG commonly experience symptoms including reduced appetite, abdominal pain, and vomiting. Given the completeness and analyzability of outcome measures data, we ultimately chose anorexia and abdominal pain as the outcome measures for TCM symptom scoring to reflect the therapeutic effects of the six interventions. In all, nine RCTs reported on this outcome indicator, including 1570 patients.

The heterogeneity test indicates statistically significant heterogeneity in the TCM symptom scores of abdominal pain and loss of appetite between studies (*P *< 0.0001, *I²*= 95.6 %, *P< *0.0001, *I²*= 95.9 %). The inconsistency test using the node partitioning method shows that the PSRF is 1, indicating convergence of the iterations well.

For abdominal pain, the results of ranking the interventions by SUCRA showed that CON (80.77 %) > CLD+CON (61.09 %) > SD+CON (45.3 %) > XLD+CON (33.43 %) > LD+CON (29.58 %). For inappetence, the results of ranking the interventions by SUCRA showed that CON (60.64 %) > SD+CON (55.50 %)> XLD+CON (44.38 %) > SBP+CON (40.29 %) > LD+CON (34.64 %) > CLD+CON (14.55 %).

The BNMA results of abdominal pain show that compare to CON, SD+CON (SMD= -0.48, 95 % CI -0.97, 0), LD+CON (SMD= -0.7, 95 % CI -1.38, -0.02), XLD+CON (SMD=-0.62, 95 % CI -1.02, -0.2) could improve symptoms of abdominal pain, CLD +CON (SMD= -0.27, 95 % CI -0.75, 0.22) showed no notable distinct in the symptoms of abdominal pain. And the results of inappetence show that compare to CON, SD+CON (SMD= -0.08, 95 % CI -2.38, 2.21), LD+CON (SMD= -0.9, 95 % CI -4.16, 2.36), XLD+CON (SMD= -0.47, 95 % CI -2.78, 1.83), SBP+CON (SMD= -0.66, 95 % CI -3.92, 2.61), CLD+CON (SMD= -1.75, 95 % CI -4.07, 0.54) showed no notable distinct in the symptoms of inappetence (Supplementary Figures 1 and 2).

The above results indicated that the group mostly using combined herbal formulations, significantly outperformed the group receiving conventional interventions in the symptoms of abdominal pain. Considering abdominal, LD+CON (rank 5) and XLD+CON (rank 4) may have a relative advantage. For inappetence, CLD+CON (rank 6) and LD+CON (rank 5) have shown potential advantages over other treatment options.

### Comprehensive therapeutic efficacy

**Figure 6:**
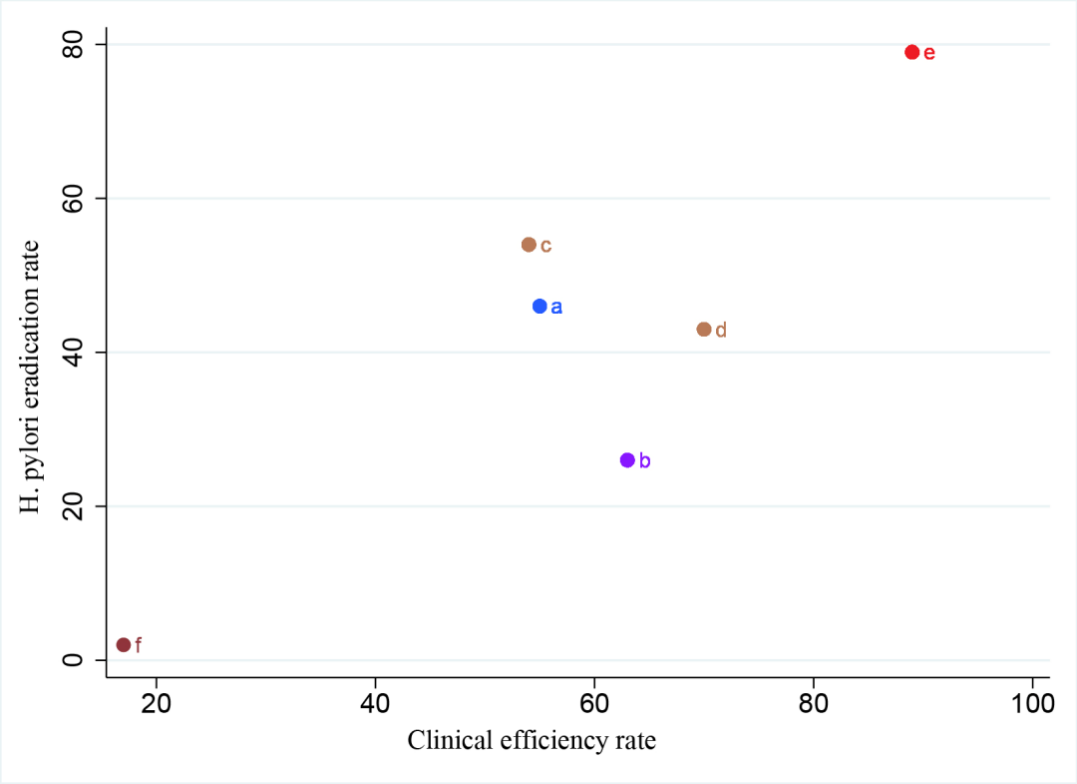
Sequential analysis of Comprehensive therapeutic efficacy. (Notes: (a) *Sijunzi *decoction + Conventional therapy. (b) *Liujunzi *decoction + Conventional therapy. (c) *Xiangsha Liujunzi* decoction + Conventional therapy. (d) *Shenling Baizhu* powder + Conventinal therapy. (e) *Chaishao Liujunzi* decoction + Conventional therapy)

In order to more intuitively reflect which strategy is optimal, we selected clinical efficacy and *H. pylori* eradication rate as indicators, assessing each intervention for its comprehensive effectiveness. The combined treatment effect of the six intervention plans could be categorized into five levels, ranging from best to worst: the first level included CLD+CON, the second level included XLD+CON and SBP + CON, the third level included SD+CON, the fourth level including SBP+CON, the fifth level included CON (Figure 6(Fig. 6)).

### Publication bias assessment and sensitivity analysis

We employed funnel plots, Egger's test, Begg's test to assess the presence of publication bias, and taking clinical efficiency indicators as representatives of publication bias. In the evaluation conducted by Egger's test, *P* < 0.001, 95 % CI (2.85, 5.05). In the Begg's test examination, Kendall's score is displayed as 189, with a standard deviation of 37.86. This study exhibited a certain degree of publication bias. After sequentially excluding each study in sensitivity analysis, we observed no significant change in the overall RR values for clinical efficacy, *H. pylori* eradication rate, and Incidence of adverse effects, thus underscoring the reliability and robustness of our results. However, the heterogeneity of Yin and Xu and Zhou is significant (Yin and Xu, 2021[70]; Xu et al.,2010[63]; Zhou et al., 2015[79]), triggering conspicuous changes in the overall effect size for the TCM symptom scores of abdominal pain and inappetence (Supplementary Figures 4-7).

## Discussion

### Summary of the evidence

This study represents the first evaluation of the efficacy and safety of SF in combination with conventional therapy for CAG. A total of 24 studies involving 2098 patients were included. The findings reveal that the combination of five Chinese herbal remedies with conventional therapies is effective. Moreover, it significantly improves clinical effectiveness and eradication rate of *H. pylori*, while alleviating Chinese medicine symptoms and reducing adverse reactions in patients with chronic gastritis.

The results of SUCRA indicated that CLD+CON is the optimal strategy among these six treatments strategies. Not only does it significantly improve the clinical efficiency (rank 1) and the *H. pylori* eradication rate (rank 1), it also reduces the incidence of adverse effects (rank 6) improving security. However, compared with other Chinese herbal formulas, CLD (rank 2) did not have a particularly strong performance in the score of TCM symptoms of abdominal pain. In contrast, LD+CON (rank 5) performs optimally in this area. But its performance in terms of clinical efficacy (rank 3), *H. pylori* eradication rate (rank 5) and incidence of adverse effects (rank 3) was not satisfactory. Furthermore, it is worth to mention that XLD+CON shows the worst performance in incidence of adverse effects. Actually, the study utilizing the intervention strategy XLD+CON assessed the incidence of adverse events. However, no adverse reactions were observed among the participants in the aforementioned study utilizing the intervention strategy XLD+CON. Consequently, the comparison between the control and experimental groups in that study lacks meaningfulness, potentially contributing to its top-ranking position. As we observed, these five traditional Chinese medicine formulas displayed significant therapeutic effects, and there were also differences among them. This is precisely the primary focus of our discussion.

### The potential mechanism of SF for the treatment of CAG

The imbalance of gastric mucosal environment is of great importance during the progression of atrophic gastritis (Liu et al., 2023[32]), with mucosal damage often being considered an early indicator (Liu et al., 2023[32]; Xu et al., 2023[62]). The damaged gastric mucosa releases numerous inflammatory mediators, attracting inflammatory cells to infiltrate and clear pathogens at the site of injury, while further activating inflammatory pathways. It is reported that the Nuclear Factor-kappa B (NF-κB), PI3K/Akt, and Hedgehog signaling pathways were significantly activated (Jiang et al., 2021[19]; Xie and Liu, 2018[60]; Yang et al., 2013[66]), leading to the overexpression of inflammatory mediators such as tumor necrosis factor-alpha (TNF-α), interleukin-6 (IL-6), IL-11, and cyclooxygenase-2 (COX-2), which further exacerbates the inflammatory response, gastric mucosal damage, and triggers dysplasia (Lenti et al. 2022[22]). It is worth mentioning that *H. pylori* infection, often linked to CAG patients (Holleczek et al. 2020[14]), disrupted gastric physiological processes by altering lysosomal membrane permeability to induce autophagy, boosting glutamine consumption and raising reactive oxygen species production (ROS), thus hastening the atrophy of gastric gland cells (Chauhan et al., 2019[5]; Ricci et al., 2014[45]). Moreover, it promoted excessive expression of IL-11, resulting in a reduction of stromal and chief cells in the gastric fundus, and weakened gastric barrier function (Buzzelli et al., 2019[3]; Howlett et al., 2012[15]). As the gastric barrier function weakened, the permeability of the gastric mucosa increased, facilitating the adherence and colonization of bacteria, viruses, and other pathogenic microorganisms (Chauhan et al., 2019[5]; Engevik et al., 2020[9]). Concurrently, the heightened sensitivity of the gastric mucosa to external stimuli prompted the increased release of inflammatory mediators and neurotransmitters (Zhang et al., 2022[73]; Zhong, 2021[78]). This escalation of inflammatory response resulted in further damage to the gastric mucosa, perpetuating a vicious cycle. Hence, alleviating mucosal damage and promoting the repair of barrier function was the key to reversing the progression of gastric mucosal lesions (Lu et al., 2022[35]; Park et al., 2021[41]). 

Encouragingly, several studies have revealed the potential of SF in anti-inflammatory and promoting the regeneration and repair of gastric mucosal tissues, which may stem from the abundant active ingredients it contains (Li et al., 2022[23]; Tian et al., 2019[52]; Zhao et al., 2002[75]). As the primary compound in ginseng, ginsenoside demonstrated anti-inflammatory properties by down-regulating the expression of pro-inflammatory molecules (TNF-α, IL-6, and COX-2) through modulation of the NF-κB and STAT3 signaling pathways (Im, 2020[18]; Kim et al., 2015[20]; To et al., 2022[53]). Our previous studies revealed that ginsenoside Rg1 down-regulated the transcription and protein expression of downstream target genes (c-myc, cyclin D1, and Birc5), thereby reversing intestinal metaplasia and partial dysplasia in gastric precancerous lesions. This effect was achieved through reducing nuclear translocation of β-catenin and disrupting β-catenin/TCF4 interactions (Zeng et al. 2021[71]). Moreover, ginsenoside was identified to preserve cellular morphology and suppress pyroptosis, thus alleviating and retarding the progression of CAG through modulation of the NF-κB/NLRP3/GSDMD pathway (Zhou et al. 2024[80]). 

Recently, another study demonstrated that costunolide effectively reversed the induction of apoptosis by MNNG in GES-1 cells, promoted the expression of Nrf2 in gastric tissue, and inhibited the expression of γ-H2AX and PARP1 in gastric tissue to suppress DNA damage and cell apoptosis, thereby alleviating damage (Wang et al. 2024[56]). Furthermore, it was also found that quercetin can significantly alleviate gastric mucosal oxidative damage, restore mitochondrial function, and repair gastric mucosal barrier by modulating the PI3K/AKT signaling pathway (Yao et al., 2021[68]). What's more, it promoted the proliferation of gastric mucosal cells and counteracted apoptosis in gastric epithelial cells induced by *H. pylori* infection by regulating the levels of BAX, BCL-2, and p38MAPK (Zhang et al., 2017[72]).

Several recent studies reported similar findings. XLD has been revealed to ameliorate gastric mucosal injury in rat with *H. pylori* infection-induced CAG by down-regulating TLR2, TLR4, P-p38mapk, NF-κB protein expression, which was associated with direct inhibition of TLR4/MAPK/NF-κB/iNOS/NO signaling pathways (Lin et al., 2016[30]). Administering water-soluble polysaccharides from *Poria cocos* to mice via gavage reduced the abundance of *H. pylori *(Zou et al., 2021[81]). Additionally, ginsenoside has been demonstrated to significantly inhibit the TLR4-MyD88-MAPK signaling pathway, thus reducing the Firmicutes/Bacteroidetes ratio and regulating the gut microbiota imbalance caused by antibiotics (Bai et al., 2021[1]). That may be the reason why the Chinese herbal formula group had a higher *H. pylori* eradication rate compared to the conventional therapy in our study.

As indicated by the meta-analysis results, CLD exhibited superiority among these five herbal formulas, which can be traced. Tu et al. explored the potential mechanisms of CLD in improving CAG by integrating network pharmacology and experimental investigations (Tu et al., 2022[54]). This study found that CLD significantly reduced the expression of IL-1β and IL-6, alleviating inflammatory responses, but also up-regulated the expression of Shh, Ptch1, and Gli1 to promote gastric mucosal cell proliferation through modulating hedgehog signaling pathway. Kaempferol was its core component. What's more, kaempferol downregulated the expression of TNF-α, IL-1β, and IL-8 by inhibiting the translocation of cytotoxin-associated gene A (CagA) and vacuolating cytotoxin A (VacA) (Qing et al., 2023[44]; Yeon et al., 2019[69]), reducing bacterial adhesion to the gastric mucosa by inhibiting urease activity as well as disrupting ATP-binding cassette proteins and fatty acid metabolism (Yang et al., 2022[67]), thus suppressing the growth and proliferation of *H. pylori*. This may be why CLD showed the best *H. pylori* eradication rates in these five herbal formulas.

It is worth emphasizing that CLD has the potential to regulate the PI3K/AKT signaling pathway. As it is well-known, the PI3K/AKT signaling pathway plays a crucial role in regulating various cellular processes such as growth, proliferation, survival, and metabolism, aberrant activation of this pathway is frequently observed in atrophic gastritis (Navaei et al., 2022[40]; Xie and Liu, 2018[60]). This aberrant activation leads to increased expression of transforming growth factor-alpha (TGF-α), followed by up-regulation of epidermal growth factor receptor (EGFR) and abnormal expression of TFF-α, disrupting the normal proliferation regulation of cells and consequently promoting the progression of precancerous lesions to gastric cancer (Chaturvedi et al., 2014[4]; Filipe et al., 1995[10]). Notably, *in vitro* experiments have shown that CLD effectively reduced inflammation induced by bile acids in gastric epithelial cells and inhibits cell proliferation. This was achieved by negatively regulating the EGFR/PI3K/AKT/mTOR pathway to reverse intestinal epithelial metaplasia (Sun et al., 2023[50]). Additionally, it suppressed the AKT/mTOR pathway, which has been demonstrated to significantly induce apoptosis in cisplatin-induced drug-resistant cells (Peng et al., 2010[42]). Saikosaponin inhibited the IKK β/NF-κB pathway, leading to apoptosis and autophagy in GC cells while increasing the sensitivity of gastric cancer cells to Cisplatin. Moreover, isorhamnetin inhibited GC cell proliferation and promoted apoptosis by modulating the PI3K/AKT pathway (Lv et al., 2024[37]). These pharmacological activities may contribute to the beneficial effects of CLD in alleviating inflammation in chronic atrophic gastritis, gastric mucosal atrophy, reversing intestinal metaplasia, and preventing carcinogenesis.

Taken together, both CON and herbal remedies have their own strengths and limitations. CON, as a conventional treatment strategy, can be more targeted and precise, especially for patients with CAG accompanied by *H. pylori* infection, which can control the infection in a short time. However, the development of drug resistance brought about by long-term use has created new challenges for this therapy (Rockhold, 2017[46]). Our results showed that Sijunzi-similar formulae display significant advantages. Concerning clinical efficiency and *H. pylori* eradication rate, CLD+CON was the most favorable treatment plan relative to other options, whereas CON demonstrated the least favorable therapeutic effects (clinical efficiency: CLD+CON (89.37 %) vs CON (16.67 %); *H. pylori* eradication rate: CLD+CON (79.14 %) vs CON (2.30 %)). Moreover, regarding TCM symptom score, CON consistently produced the least favorable outcomes (abdominal pain: CON (80.77 %) vs LD+CON (29.58 %); inappetence: CON (60.64 %) vs CLD+CON (14.55 %)). The comprehensive therapeutic effects of CLD are the most prominent ones. Therefore, CLD may represent an optimal and complementary therapeutic strategy.

### Limitations

Firstly, it should be noted that the included studies have methodological limitations. We evaluated 24 randomized controlled trials using Review Manager 5.3 software. Most studies exhibited unclear biases, particularly selection and performance biases. None of the included RCTs mentioned whether participant blinding, allocation concealment, and staff blinding were implemented. Undoubtedly, this has somewhat compromized the credibility and effectiveness of the trial results. Furthermore, the included studies only assessed the effects of these six treatment regimens at the end of the trials, without tracking symptoms, signs, and quality of life of patients after the trial. This lack of follow-up prevents us from understanding the recurrence status of CAG patients after treatment with these six interventions. Moreover, one characteristic of individualized traditional Chinese medicine treatment is that even studies of the same intervention exhibit differences in treatment regimens (duration, frequency of medication) and the drugs used (types and dosages), which have been overlooked. Additionally, the exploration of the mechanism of action of traditional Chinese medicine formulas in treating CAG remains incomplete. On one hand, formulae consist of various Chinese herbs with diverse and complex components, necessitating more sophisticated methods to explore the types of these components, as well as their targets and pathways. On the other hand, the occurrence and development of CAG are highly complex, with most studies only investigating the mechanisms of action of these five traditional Chinese medicine formulas and their active ingredients against atrophic gastritis induced by *H. pylori*, bile acids, and nitrosamines, lacking comprehensive evaluation of their efficacy. Therefore, in the future, we need to conduct more rigorous and comprehensive clinical trials to provide more compelling evidence.

## Conclusion

Based on the BNMA results, combining herbal formulation with traditional treatment demonstrated superior clinical efficacy, *H. pylori* eradication rate, TCM symptom score, and lower incidence of adverse effects compared to traditional therapy alone. Among the six strategies evaluated, CLD+CON was identified as the optimal treatment approach. However, the lack of diversity in the study designs limited the ability to directly compare different herbal formula combinations, reducing the reliability of the results. It is imperative to conduct an increased number of meticulously designed RCTs to explore the effectiveness of combining herbal formulae with conventional treatment strategies for patients with CAG.

## Notes

Meilan Huang, Shiman Luo and Jiayue Yang contributed equally as first author.

Xiao Ma, Jinhao Zeng (Department of Gastroenterology, Hospital of Chengdu University of Traditional Chinese Medicine, Chengdu 610072, China; E-mail: zengjinhao@cdutcm.edu.cn) and Thomas Efferth (Department of Pharmaceutical Biology, Institute of Pharmaceutical and Biomedical Sciences, Johannes Gutenberg University, 55128 Mainz, Germany; E-mail: efferth@uni-mainz.de) contributed equally as corresponding author.

## Declaration

### Author contribution

Meilan Huang: Conceptualization, methodology, data curation, visualization, writing-review & editing. Shiman Luo: Data curation, visualization, writing - original draft. Jiayue Yang, Huiling Xiong and Xiaohua Lu: Data curation, visualization, methodology. Thomas Efferth (corresponding author) and Jinhao Zeng (corresponding author): Conceptualization, visualization, writing-review & editing. Xiao Ma (Corresponding author): Conceptualization, visualization, writing-review & editing.

### Funding

This work was supported by the National Natural Science Foundation of China (82174346), the Major Scientific Research Problems and Key Topic of Medical Technology Problems of China Medical Education Association (2022KT016), the Science and Technology Project of Sichuan Province (2023NSFSC0039 and 2023NSFSC0687), “Hundred Talents Program” of the hospital of the Chengdu University of Traditional Chinese Medicine (22-B09), and the Xinglin Scholar Research Project of Chengdu University of TCM (QJJJ2022010 and QJRC2022028). 

### Declaration of interest conflicts

All authors declare no conflict of interest related to the work and adhere to the principles of integrity and objectivity to ensure the neutrality and reliability of the research results.

### Acknowledgments

The authors are grateful to the reviewers and to the authors of all the references.

### Data availability statement

The datasets used and analyzed in this study are available upon request from the respective authors. All supplementary materials have been provided.

## Supplementary Material

Supplementary information

## Figures and Tables

**Table 1:**
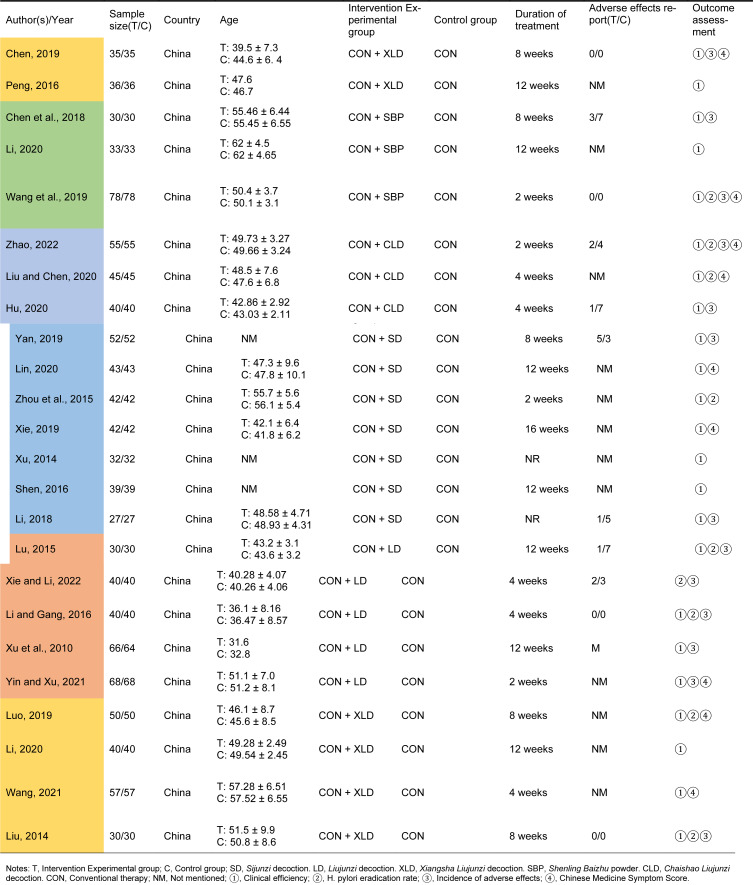
The characteristics of included studies
