# Supplementary material for: Optimized therapeutic potential of Sijunzi-similar formulae for chronic atrophic gastritis via Bayesian network meta-analysis
Source: EXCLI J. 2024 Sep 6;23:1185–207. doi: 10.17179/excli2024-7618 (PMC11484511; doi:10.17179/excli2024-7618)
Supplement: Supplementary information [file EXCLI-23-1185-s-001.pdf]

EXCLI Journal 2024;23:1185-1207 – ISSN 1611-2156
Received: July 10, 2024, accepted: August 29, 2024, published: September 06, 2024

Supplementary information to:

OPTIMIZED THERAPEUTIC POTENTIAL OF SIJUNZI-SIMILAR FORMULAE FOR CHRONIC ATROPHIC GASTRITIS VIA BAYESIAN NETWORK META-ANALYSIS

Meilan Huang[a,b,1], Shiman Luo[d,1], Jiayue Yang[a,1], Huiling Xiong[d], Xiaohua Lu[e], Xiao Ma[d,e*], Jinhao Zeng[a,b,c,*], Thomas Efferth[e*]

[a] Department of Gastroenterology, Hospital of Chengdu University of Traditional Chinese Medicine, Chengdu 610072, China
[b] School of Clinical Medicine, Chengdu University of Traditional Chinese Medicine, Chengdu 610075, China
[c] TCM Regulating Metabolic Disease Key Laboratory of Sichuan Province, Hospital of Chengdu University of Traditional Chinese Medicine, Chengdu 610072, China
[d] State Key Laboratory of Southwestern Chinese Medicine Resources, School of Pharmacy, Chengdu University of Traditional Chinese Medicine, Chengdu 611137, China
[e] Department of Pharmaceutical Biology, Institute of Pharmaceutical and Biomedical Sciences, Johannes Gutenberg University, 55128 Mainz, Germany

[1] Co-first authors
[*] **Corresponding authors:** Xiao Ma, State Key Laboratory of Southwestern Chinese Medicine Resources, School of Pharmacy, Chengdu University of Traditional Chinese Medicine, Chengdu, 611137, China.
E-mail: tobymaxiao@cdutcm.edu.cn
Jinhao Zeng, Department of Gastroenterology, Hospital of Chengdu University of Traditional Chinese Medicine, Chengdu 610072, China.
E-mail: zengjinhao@cdutcm.edu.cn
Thomas Efferth, Department of Pharmaceutical Biology, Institute of Pharmaceutical and Biomedical Sciences, Johannes Gutenberg University, 55128 Mainz, Germany.
E-mail: efferth@uni-mainz.de

https://dx.doi.org/10.17179/excli2024-7618

This is an Open Access article distributed under the terms of the Creative Commons Attribution License (http://creativecommons.org/licenses/by/4.0/).

EXCLI Journal 2024;23:1185-1207 – ISSN 1611-2156
Received: July 10, 2024, accepted: August 29, 2024, published: September 06, 2024

Supplementary Table 1: The composition of Chinese herbal formulas

Formulas name	Composition	Ratio	Main active ingredients
**Sijunzi Decoction**	*Panax ginseng* C. A. Mey.	1	ginsenoside, volatile oil, polysaccharide
	Poria cocos (Schw.) Wolf	1	polysaccharide
	Glycyrrhiza uralensis Fisch.	1	flavonoid, polysaccharide
	Atractylodes macrocephala Koidz.	1	polysaccharide, lactone
**Liujunzi Decoction**	*Panax ginseng* C. A. Mey.	2	ginsenoside, volatile oil, polysaccharide
	Poria cocos (Schw.) Wolf	2	polysaccharide
	Glycyrrhiza uralensis Fisch.	2	flavonoid, polysaccharide
	Atractylodes macrocephala Koidz.	3	polysaccharide, lactone
	Pinellia ternata (Thunb.) Breit.	3	alkaloid, sterol
	Citrus reticulata Blanco	2	volatile oil
**Xiangsha Liujunzi Decoction**	*Panax ginseng* C. A. Mey.	1	ginsenoside, volatile oil, polysaccharide
	Poria cocos (Schw.) Wolf	2	polysaccharide
	Glycyrrhiza uralensis Fisch.	1	flavonoid, polysaccharide
	Atractylodes macrocephala Koidz.	2	polysaccharide, lactone
	Pinellia ternata (Thunb.) Breit.	1	alkaloid, sterol
	Citrus reticulata Blanco	1	volatile oil
	Aucklandia lappa Decne.	1	lactone, alkaloid
	Amomum villosum Lour.	1	volatile oil
**Shenling Baizhu Powder**	*Panax ginseng* C. A. Mey.	2	ginsenoside, volatile oil, polysaccharide
	Poria cocos (Schw.) Wolf	2	polysaccharide
	Glycyrrhiza uralensis Fisch.	2	flavonoid, polysaccharide
	Atractylodes macrocephala Koidz.	2	polysaccharide, lactone
	Dolichos lablab L.	1	polysaccharide
	Dioscorea opposita Thunb.	2	polysaccharide, saponin
	Nelumbo nucifera Gaertn.	1	alkaloid, flavonoid
	Coix lacryma-jobi L.var.*mayuen*(Roman.) Stapf	1	polysaccharide
	Amomum villosum Lour.	1	volatile oil
	Platycodon grandiflorus (Jacq.) A. DC.	1	saponin, flavonoid, poly-saccharide
**Chaishao Liujunzi Decoction**	*Panax ginseng* C. A. Mey.	3	ginsenoside, volatile oil, polysaccharide
	Poria cocos (Schw.) Wolf	2	polysaccharide
	Glycyrrhiza uralensis Fisch.	1	flavonoid, polysaccharide
	Atractylodes macrocephala Koidz.	2	polysaccharide, lactone
	Pinellia ternata (Thunb.) Breit.	2	alkaloid, sterol
	Citrus reticulata Blanco.	2	volatile oil
	Bupleurum chinense DC.	2	saponin, polysaccharide
	Paeonia lactiflora Pall.	3	Paeoniflorin

EXCLI Journal 2024;23:1185-1207 – ISSN 1611-2156
Received: July 10, 2024, accepted: August 29, 2024, published: September 06, 2024

Supplementary Table 2: The search strategy for English databases

Database	Number	Search terms
**EMBASE**	#1	chronic atrophic gastritis':kw AND 'sijunzi decoction chronic atrophic gastritis':kw OR 'liujunzi decoction chronic atrophic gastritis':kw OR 'xiangsha liujun decoction chronic atrophic gastritis':kw OR 'shenling baizhu powder orchronic atrophic gastritis':kw OR 'chaishao liujunzi decoction orchronic atrophic gastritis':kw
	#2	(('sijunzi'/exp OR sijunzi) AND ('decoction'/exp OR decoction) AND chronic AND atrophic AND ('gastritis'/exp OR gastritis))) OR'liujunzi decoction chronic atrophic gastritis' OR (('liu-junzi'/exp OR liujunzi) AND (''decoction'/exp OR decoction) AND chronic AND atrophic AND ('gastritis'/exp OR gastri-tis))OR 'xiangsha liujun decoction chronic atrophic gastritis' OR (xiangsha AND liujun AND ('decoction'/exp OR decoction) AND chronic AND atrophic AND ('gastritis'/exp OR gastritis)) OR 'shenling baizhu powder orchronic atrophic gastritis' OR (shenling AND (''baizhu'/exp OR baizhu) AND (''powder'/exp OR powder) AND orchronic AND atrophic AND ('gastritis'/exp OR gastritis))OR'chaishao liujunzi decoction chronic atrophic gastritis' OR ((''chaishao'/exp OR liujunzi) AND (''decoc-tion'/exp OR decoction) AND chronic AND atrophic AND ('gas-tritis'/exp OR gastritis)))AND ('chronic atrophic gastritis':kw AND 'sijunzi decoction chronic atrophic gastritis':kw OR 'liu-junzi decoction chronic atrophic gastritis':kw OR 'xiangsha liu-jun decoction chronic atrophic gastritis':kw OR 'shenling baizhu powder orchronic atrophic gastritis':kw OR 'chaishao liujun decoction chronic atrophic gastritis':kw)
	#3	('chronic atrophic gastritis'/exp OR 'chronic atrophic gastritis') AND ('sijunzi decoction'/exp OR 'sijunzi decoction') OR 'liu-junzi decoction'/exp OR 'liujunzi decoction' OR 'xiangsha liujun decoction' OR 'shenling baizhu powder'/exp OR 'shenling baizhu powder' OR 'chaihu liu junzi decoction'
	#4	#1 OR #2 OR #3
**PubMed**	#1	"Gastritis, Atrophic"[Mesh] OR (Atrophic Gastritides) OR (Atrophic Gastritis) OR (Gastritides, Atrophic)
	#2	(Sijunzi decoction) [Supplementary Concept] OR (SJZD) OR (Sijunzi formula) OR (liujunzi decoction) [Supplementary Con-cept] OR (xiangsha liujunzi decoction) [Supplementary Con-cept] OR OR (shenling baizhu powder） [Supplementary Concept] OR (chaihshaoliujunzi decoction) [Supplementary Concept]
	#3	#1 AND #2
**Web of science**	#1	(TS=((Gastritis, Atrophic) OR (Atrophic Gastritides) OR (Atrophic Gastritis) OR (Gastritides, Atrophic))) AND TS=(sijunzi decoction OR SJZD OR sijunzi formula OR liujunzi decoction OR xiangsha liujun decoction OR Shenling Baizhu Powder OR Chaishao Liujunzi Decoction)

EXCLI Journal 2024;23:1185-1207 – ISSN 1611-2156
Received: July 10, 2024, accepted: August 29, 2024, published: September 06, 2024

**Cochrane Library**	#1	MeSH descriptor: [Gastritis, Atrophic] this term only
	#2	(Gastritis, Atrophic or Atrophic Gastritides or Atrophic Gastritis or Gastritides, Atrophic)
	#3	#1 OR #2
	#4	sijunzi decoction or liujunzi decoction or xiangsha liujun decoction or Shenling Baizhu Powder or chaishao liujunzi decoction
	#5	#3 AND #4

EXCLI Journal 2024;23:1185-1207 – ISSN 1611-2156
Received: July 10, 2024, accepted: August 29, 2024, published: September 06, 2024

Supplementary Table 3: List of abbreviations

Abbreviation	Full name
**BNMA**	Bayesian network meta-analysis
**CAG**	Chronic atrophic gastritis
**CagA**	Cytotoxin-associated gene A
**Cis**	Confidence intervals
**CLD**	Chaishao Liujunnzi Decoction
**COX-2**	Cyclooxygenase-2
**EGFR**	Epidermal growth factor receptor
**H. pylori**	Helicobacter pylori
**HOS-1**	Heme oxygenase-1
**IL**	Interleukin
**LD**	Liujunzi Decoction
**NF-κB**	Nuclear Factor- kappa B
**PRISMA**	Systematic Reviews and Meta-Analyses
**PSRF**	The potential scale reduction factor
**ROS**	Reactive oxygen species
**RR**	Relative risk
**SBP**	Shenling Baizhu Powder
**SD**	Sijunzi Decoction
**SF**	Sijunzi-Similar Formulae
**SMD**	Standard mean difference
**SUCRA**	The surface under the probability cumulative ranking curve
**TGF-α**	Transforming growth factor- alpha
**TNF-α**	Tumor Necrosis Factor-alpha
**VacA**	Vacuolating cytotoxin A
**XLD**	Xiangsha Liujunzi Decoction

EXCLI Journal 2024;23:1185-1207 – ISSN 1611-2156
Received: July 10, 2024, accepted: August 29, 2024, published: September 06, 2024

A.

B.

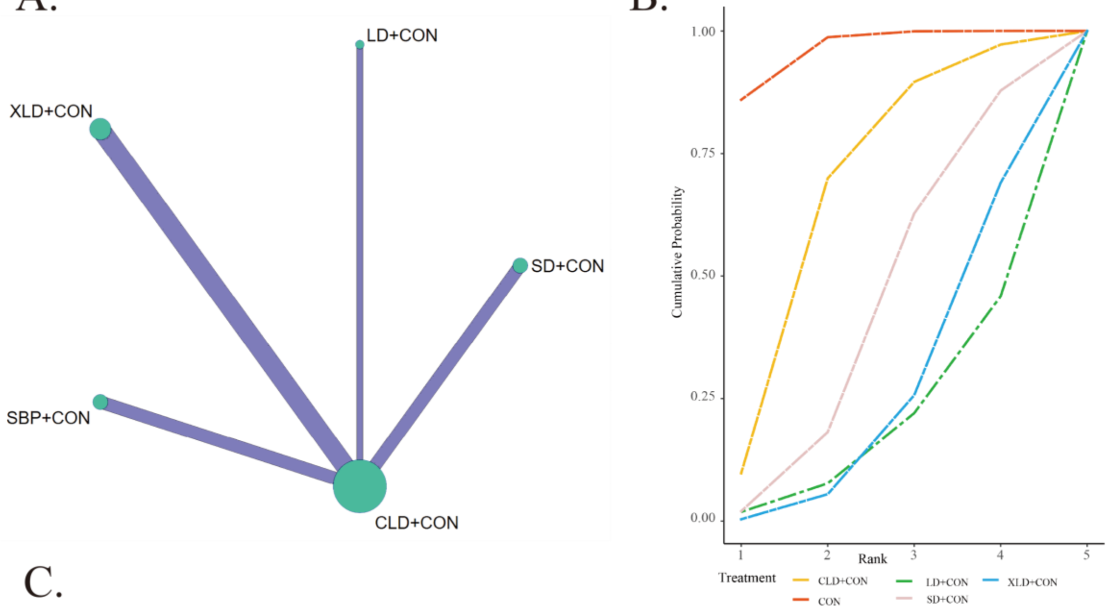

C.

SD + CON				
0.22 (-0.62, 1.05)	LD + CON			
0.14 (-0.5, 0.76)	-0.08 (-0.89, 0.71)	XLD + CON		
-0.21 (-0.91, 0.47)	-0.43 (-1.28, 0.4)	-0.35 (-0.99, 0.29)	CLD + CON	
-0.48 (-0.97, 0)	-0.7 (-1.38, -0.02)	-0.62 (-1.02, -0.2)	-0.27 (-0.75, 0.22)	CON

Supplementary Figure 1: The Bayesian Network Meta analysis of abdominal pain symptom score. A: Network comparisons of abdominal pain symptom score. **B:** The cumulated probability rank of abdominal pain symptom score. **C:** League table of Bayesian network meta-analysis for abdominal pain symptom score. (Notes: SD, Sijunzi Decoction. LD, Liujunzi Decoction. XLD, Xiangsha Liujunzi Decoction. CLD, Chaishao Liujunzi Decoction. CON, Conventional therapy)

EXCLI Journal 2024;23:1185-1207 – ISSN 1611-2156
Received: July 10, 2024, accepted: August 29, 2024, published: September 06, 2024

A.

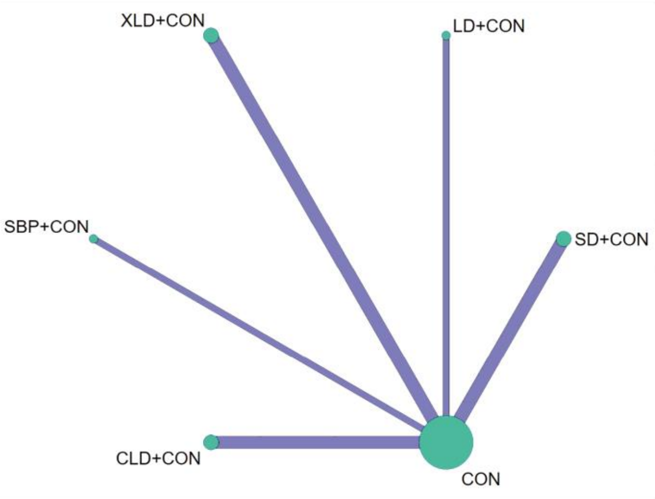

B.

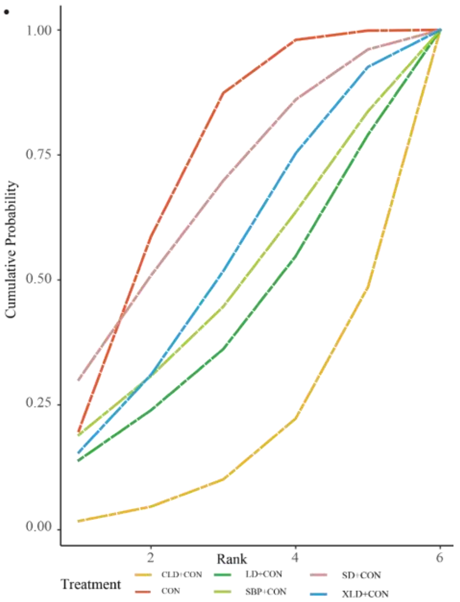

C.

SD + CON					
0.83 (-3.16, 4.8)	LD + CON				
0.4 (-2.86, 3.65)	-0.43 (-4.41, 3.55)	XLD + CON			
0.58 (-3.4, 4.57)	-0.24 (-4.84, 4.34)	0.19 (-3.81, 4.19)	SBP + CON		
1.67 (-1.57, 4.95)	0.85 (-3.13, 4.85)	1.28 (-1.96, 4.54)	1.09 (-2.89, 5.13)	CLD + CON	
-0.08 (-2.38, 2.21)	-0.9 (-4.16, 2.36)	-0.47 (-2.78, 1.83)	-0.66 (-3.92, 2.61)	-1.75 (-4.07, 0.54)	CON

Supplementary Figure 2: The Bayesian Network Meta analysis of inappetence symptom score. A: Network comparisons of inappetence symptom score. **B:** The cumulated probability rank of inappetence symptom score. **C:** League table of Bayesian network meta-analysis for inappetence symptom score. (Notes: SD, Sijunzi Decoction. LD, Liujunzi Decoction. XLD, Xiangsha Liujunzi Decoction. SBP, Shenling Baizhu Powder. CLD, Chaishao Liujunzi Decoction. CON, Conventional therapy)

EXCLI Journal 2024;23:1185-1207 – ISSN 1611-2156
Received: July 10, 2024, accepted: August 29, 2024, published: September 06, 2024

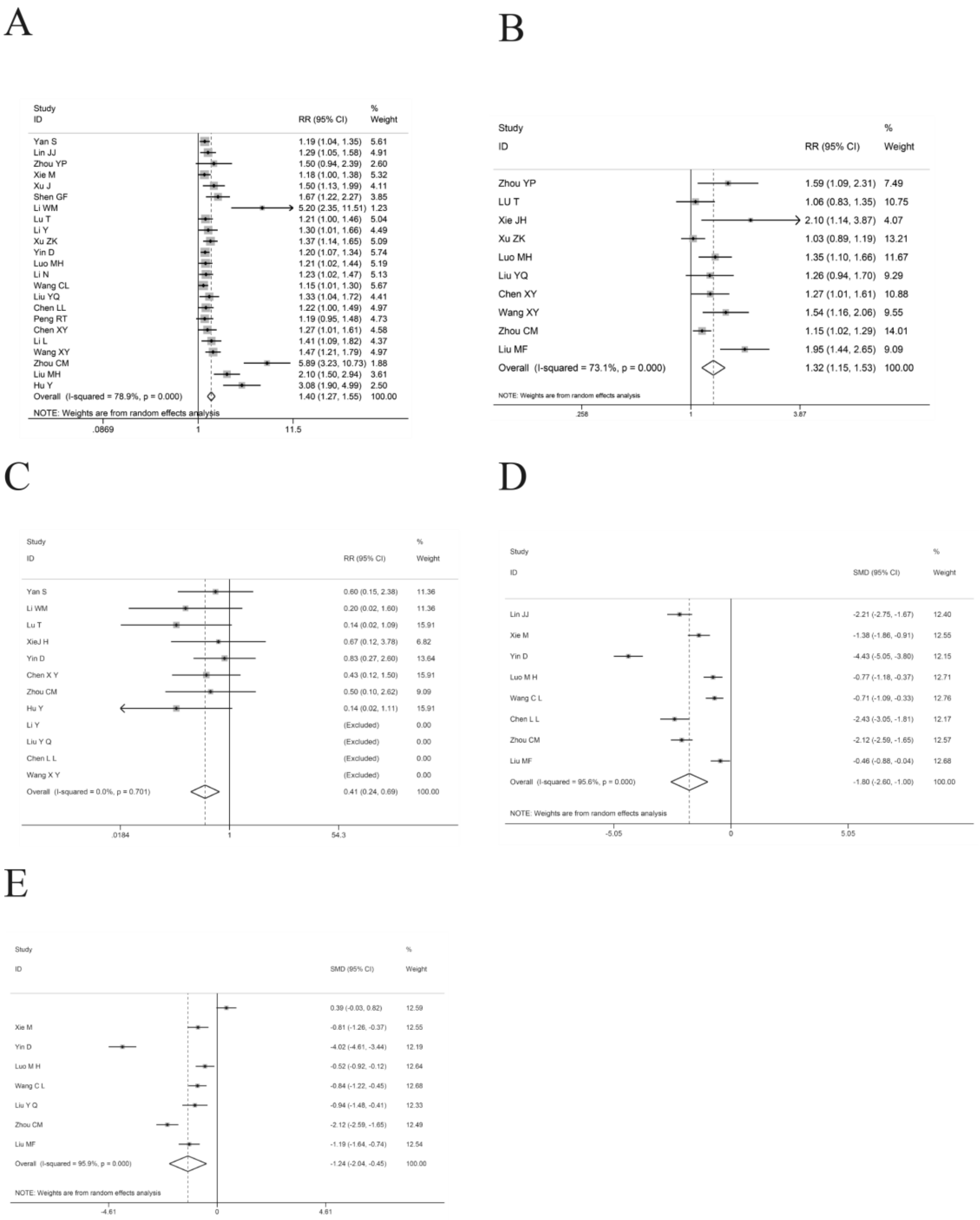

Supplementary Figure 3: Forest plot (A) Clinical efficacy, **(B)** *H. pylori* eradication rate, **(C)** Incidence of adverse effects, **(D)** The TCM symptom scores of abdominal pain, **(E)** The TCM symptom scores of inappetence

EXCLI Journal 2024;23:1185-1207 – ISSN 1611-2156
Received: July 10, 2024, accepted: August 29, 2024, published: September 06, 2024

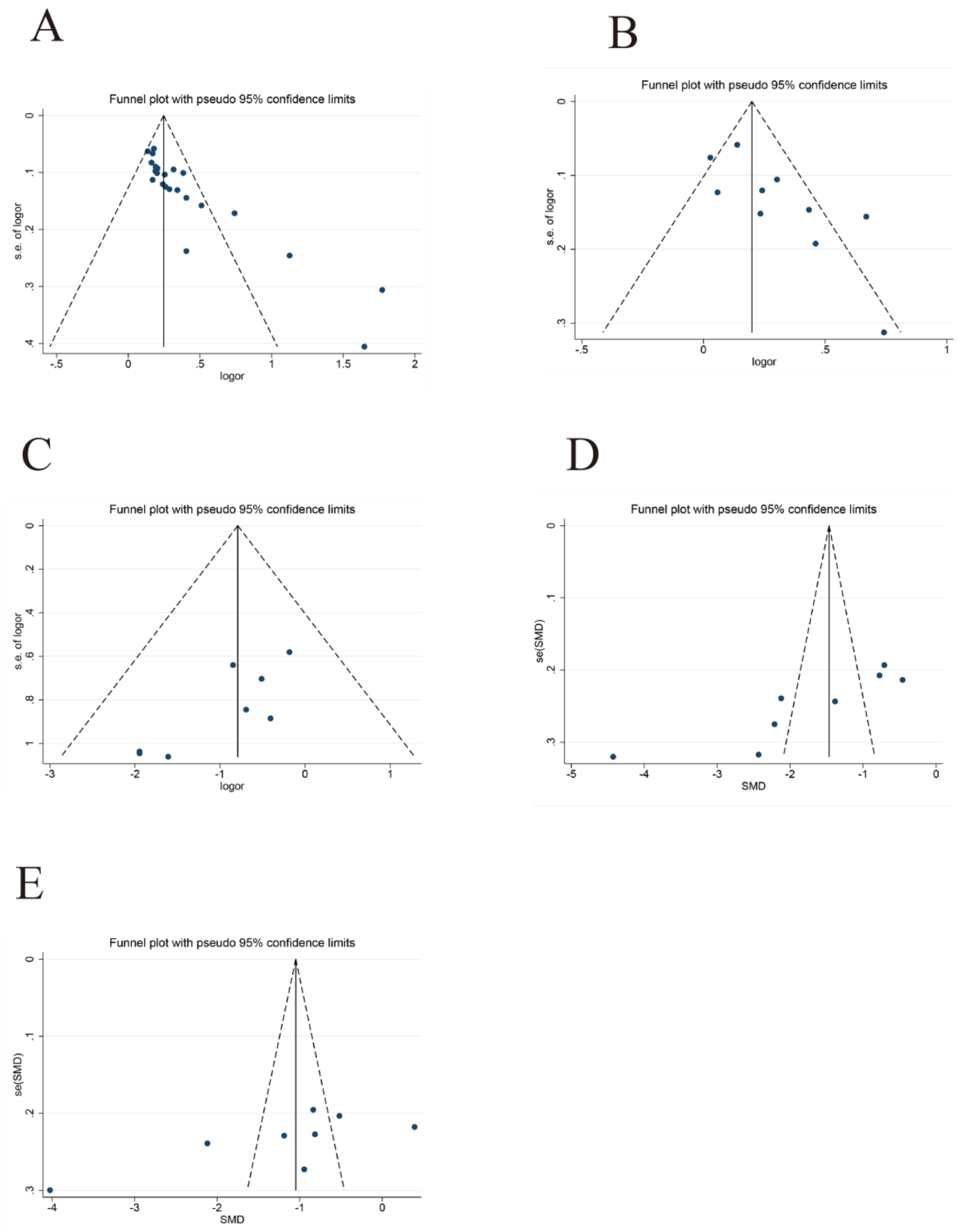

Supplementary Figure 4: Funnel plots: **(A)** Clinical efficacy, **(B)** *H. pylori* eradication rate, **(C)** Incidence of adverse effects, **(D)** The TCM symptom scores of abdominal pain, **(E)** The TCM symptom scores of inappetence

EXCLI Journal 2024;23:1185-1207 – ISSN 1611-2156
Received: July 10, 2024, accepted: August 29, 2024, published: September 06, 2024

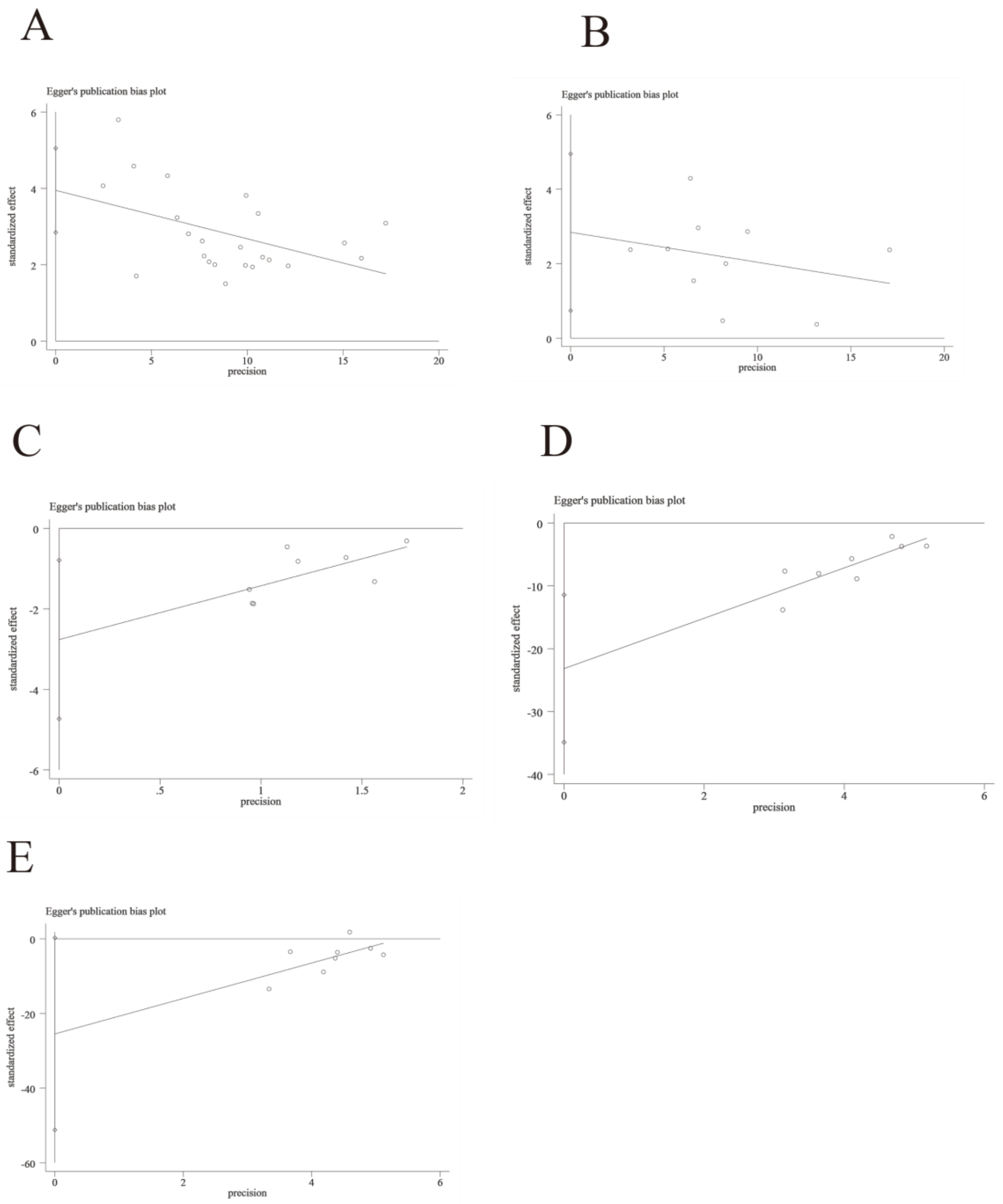

Supplementary Figure 5: Egger's publication bias plot: (A) Clinical efficacy, **(B)** *H. pylori* eradication rate, **(C)** Incidence of adverse effects, **(D)** The TCM symptom scores of abdominal pain, **(E)** The TCM symptom scores of inappetence

Received: July 10, 2024, accepted: August 29, 2024, published: September 06, 2024

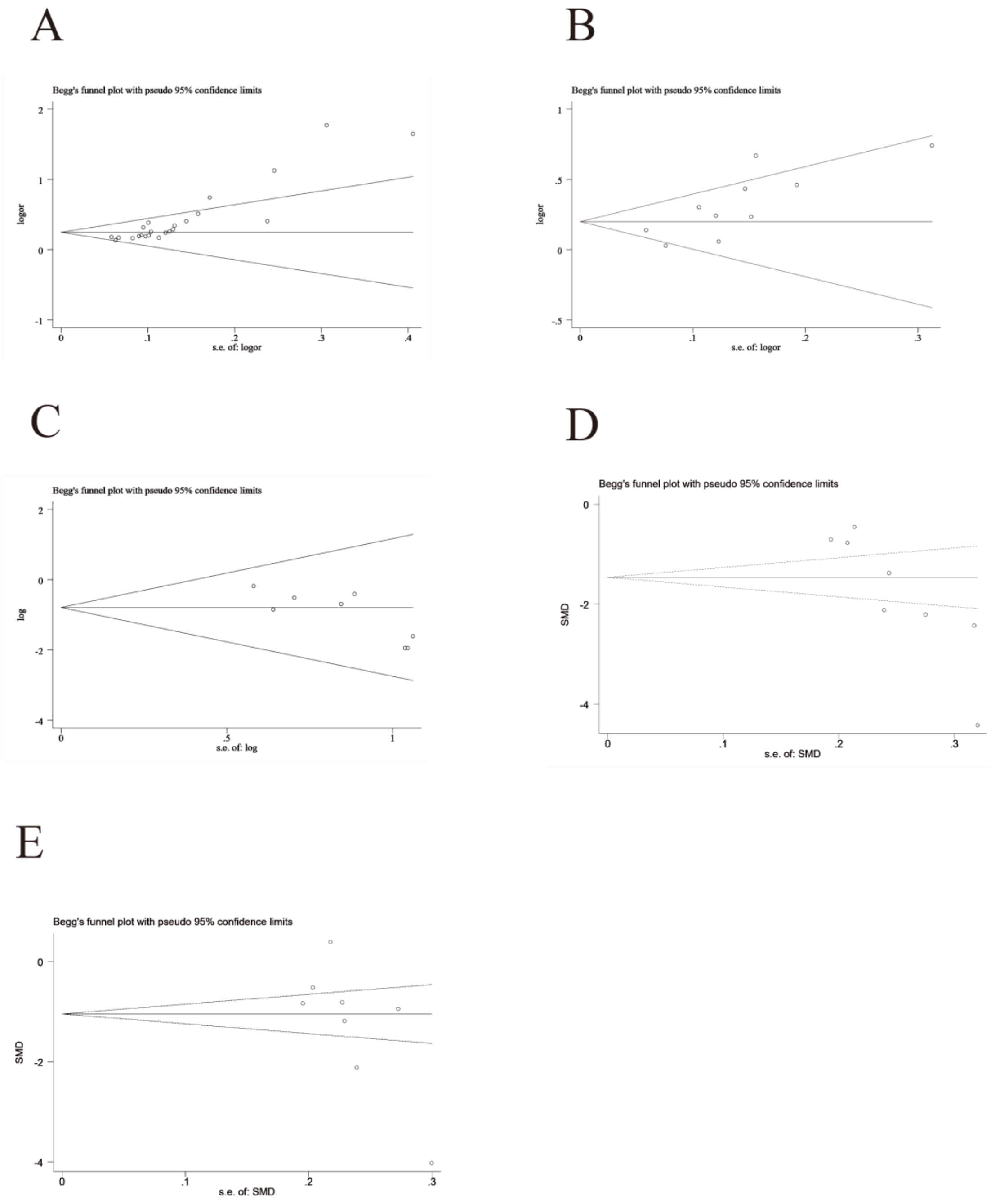

Supplementary Figure 6: Begg test plot: (A) Clinical efficacy, **(B)** *H. pylori* eradication rate, **(C)** Incidence of adverse effects, **(D)** The TCM symptom scores of abdominal pain, **(E)** The TCM symptom scores of inappetence

EXCLI Journal 2024;23:1185-1207 – ISSN 1611-2156
Received: July 10, 2024, accepted: August 29, 2024, published: September 06, 2024

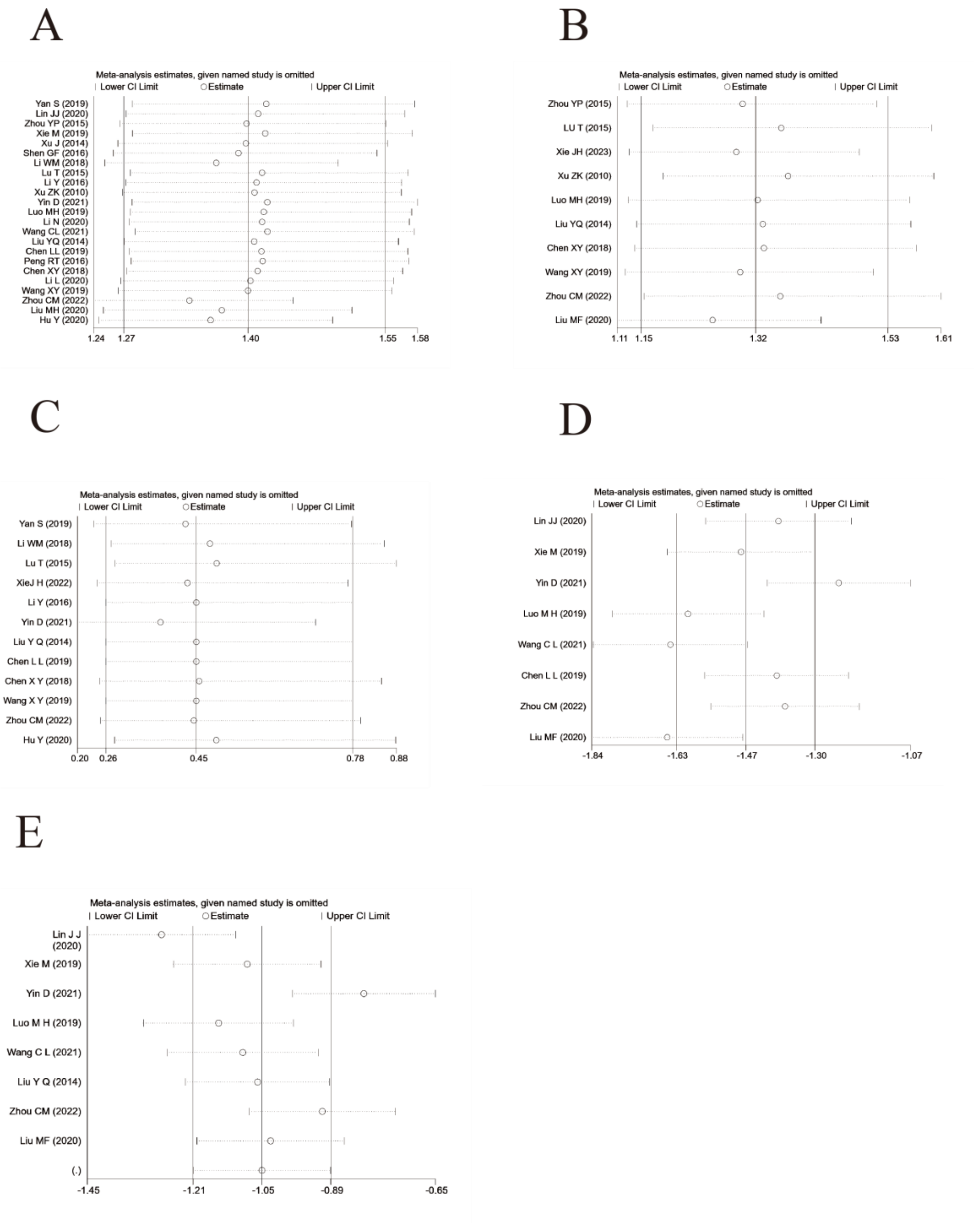

Supplementary Figure 7: Sensitive analysis (A) Clinical efficacy, **(B)** *H. pylori* eradication rate, **(C)** Incidence of adverse effects, **(D)** The TCM symptom scores of abdominal pain, **(E)** The TCM symptom scores of inappetence
